# COVID‐19 and the peripheral nervous system. A 2‐year review from the pandemic to the vaccine era

**DOI:** 10.1111/jns.12482

**Published:** 2022-03-14

**Authors:** Arens Taga, Giuseppe Lauria

**Affiliations:** ^1^ Department of Neurology Johns Hopkins University School of Medicine Baltimore Maryland USA; ^2^ Department of Clinical Neurosciences Fondazione IRCCS Istituto Neurologico “Carlo Besta” Milan Italy; ^3^ Department of Medical Biotechnology and Translational Medicine University of Milan Milan Italy

**Keywords:** CIDP, cranial neuropathy, critical illness, Guillain‐Barré syndrome, long COVID, mononeuritis multiplex, myopathy, SARS‐CoV‐2, neuralgic amyotrophy, vaccination

## Abstract

Increasing literature has linked COVID‐19 to peripheral nervous system (PNS) diseases. In addition, as we move from the pandemic to the vaccination era, literature interest is shifting towards the potential association between COVID‐19 vaccines and PNS manifestations. We reviewed published literature on COVID‐19, COVID‐19 vaccines and PNS manifestations between 1 January 2020 and 1 December 2021. For Guillain‐Barré syndrome (GBS), isolated cranial neuropathy (ICN) and myositis associated with COVID‐19, the demographic, clinical, laboratory, electrophysiological and imaging features were included in a narrative synthesis. We identified 169 studies on COVID‐19‐associated complications, including 63 papers (92 patients) on GBS, 29 papers (37 patients) on ICN and 11 papers (18 patients) on myositis. Additional clinical phenotypes included chronic inflammatory demyelinating polyneuropathy, vasculitic neuropathies, neuralgic amyotrophy, critical care‐related complications, and myasthenia gravis. PNS complications secondary to COVID‐19 vaccines have been reported during randomized clinical trials, in real‐world case reports, and during large‐scale surveillance programs. These mainly include cases of GBS, Bell's palsy, and cases of neuralgic amyotrophy. Based on our extensive review of the literature, any conclusion about a pathophysiological correlation between COVID‐19 and PNS disorders remains premature, and solely supported by their temporal association, while epidemiological and pathological data are insufficient. The occurrence of PNS complications after COVID‐19 vaccines seems limited to a possible higher risk of facial nerve palsy and GBS, to a degree that widespread access to the ongoing vaccination campaign should not be discouraged, while awaiting for more definitive data from large‐scale surveillance studies.

## 1 INTRODUCTION

An increasing body of literature, including cohort studies,^1^, ^2^, ^3^, ^4^, ^5^, ^6^, ^7^, ^8^, ^9^ has linked COVID‐19 to the development peripheral nervous system (PNS) diseases. However, findings are divergent due to methodological differences and largely variable sample sizes. Few studies used a prospective design^8^, ^10^ and focused on defined diagnoses rather than symptoms alone.^5^ Some studies relied on self‐administered questionnaires and others on hospital records, and in some cases, the diagnosis was not confirmed by neurologists.^8^ Individual diagnoses were not always supported by laboratory, electrodiagnostic (EDX), and pathology findings. As a result, PNS involvement was quite variable, ranging from 1.3% to 9.5% of cases^4^, ^8^ if individual diagnoses were considered (eg. neuropathy, myopathy, etc.), and up to 70.2% if individual symptoms were included (eg. myalgia, paresthesia, etc.).^3^

In a very large retrospective cohort including 1760 COVID‐19 patients from a single epidemic hotspot (Bergamo, Italy),^6^ 31 patients were diagnosed with PNS diseases (1.8%), including Guillain‐Barré syndrome (GBS; 17 cases), critical illness myopathy and neuropathy (nine cases), brachial plexopathy (two cases), and polyneuropathy (three cases). In a very large prospective cohort from another epidemic hotspot (New York, USA),^8^ there were 59 cases of PNS involvement among 4491 hospitalized COVID‐19 patients (1.3%) including neuropathy (35 cases), myopathy (21 cases), and GBS (three cases).

Here, we present a comprehensive narrative on 169 studies published between 1 January 2020 and 1 December 2021 on PNS involvement. Our main aims were investigating the association between COVID‐19 and PNS diseases and understanding whether COVID‐19 had any clinically meaningful impact on clinical presentation, diagnosis, and therapeutic approaches. Furthermore, as we are moving from the pandemic to the vaccination era, we provide an overview of the potential association between COVID‐19 vaccines and PNS diseases discussing the findings reported so far.

## 2 METHODS

Given the extent and heterogeneity of the topics reviewed in this paper, we aimed to provide a synthetic albeit comprehensive narrative on the published literature. However, our approach was not meant to be systematic, as commonly defined by Cochrane and PRISMA statements. A systematic approach has been attempted in the past, during the early and later stages of the pandemic, but on GBS cases only.^11^, ^12^ The data we extracted therefore were not incorporated in a meta‐analysis, but were instead the basis for our expert opinion commentaries.

We searched MEDLINE through PubMed, Web of Science and Cochrane library databases, and Google Scholar database. The search strategy included the terms (“Coronavirus” OR “Coronavirus disease” OR “novel coronavirus” OR “Severe acute respiratory syndrome coronavirus 2” OR “COVID‐19” OR “nCoV 2019” OR “SARS‐CoV‐2” OR “Long COVID” OR “COVID vaccine” OR “BNT‐162b2” OR “Pfizer” OR “mRNA‐1273” OR “Moderna” OR “Ad26.COV2.S" or “Johnson&Johnson” OR “ChAdOx1” OR “AstraZeneca” OR “Vaxzevria”) AND (“peripheral nervous systems" or “PNS” or “Guillain‐Barré syndrome” OR “GBS” OR “Miller Fisher syndrome” OR “MFS” OR “acute inflammatory demyelinating polyneuropathy” OR “AIDP” OR “acute motor axonal neuropathy” OR “AMAN” OR “acute motor sensory axonal neuropathy” OR “AMSAN”, OR “chronic inflammatory demyelinating polyneuropathy”, OR “CIDP”, OR “nerve” OR “neuropathy” OR “cranial neuropathy” OR “Bell's palsy” OR “neuritis” OR “vasculitis” OR “polyneuropathy” OR “multineuritis” OR “neuralgic amyotrophy “OR “Parsonage Turner Syndrome” OR “plexus” OR “small fiber neuropathy” OR “dysautonomia” OR “postural orthostatic tachycardia syndrome” OR “POTS” OR “muscle” OR “myopathy” OR “myositis” OR “dermatomyositis” OR “myasthenia gravis" OR “MG” OR “neuromuscular junction” OR “critical illness myopathy” OR “critical illness polyneuropathy”.

We restricted our search to peer‐reviewed studies, published in English, and importantly, to papers published between 1 January 2020 and 1 December 2021.

### 2.1 Pathophysiological insights into PNS involvement

The causal association between COVID‐19 and nervous system manifestations has been solely inferred from their temporal co‐occurrence. Two patterns have been described: (a) neurological complications occurring together with COVID‐19 symptoms and suggesting a direct viral mechanism (“para‐infectious" hypothesis), such as neuroinvasion; (b) neurological complications developing after the initial infectious symptoms and supporting indirect mechanisms (“post‐infectious" hypothesis), likely immune‐mediated.

The ability of SARS‐CoV‐2 to invade the nervous systems has been conjectured based on the known neuroinvasive capabilities, both in vivo and in vitro, of SARS‐CoV and MERS‐CoV, with whom the etiological agent of COVID‐19 (ie, SARS‐Cov‐2) has 79.5% and 50% gene homology, respectively.^13^ Given the early occurrence of anosmia and ageusia, one hypothesis is that olfactory, trigeminal, or gustative terminals could be entry routes for the virus, which could then spread to the central nervous system (CNS) through retrograde axonal transport and trans‐synaptic transfer.^14^ Lower cranial nerves could be additional entry points, causing early lower brain stem involvement and possibly explaining some peculiar features of COVID‐19, such as hypoxia out‐of‐proportion to dyspnea and the frequent occurrence of syncope.^15^ Alternative mechanisms of neuroinvasion that could apply both to the CNS and PNS include entry through circulating immune cells, infection of the vascular endothelium or crossing of the blood‐brain barrier or of the blood‐nerve barrier.^14^ A wealth of studies, including case reports and case series^16^ (Tables 1 and 2), failed to isolate SARS‐CoV‐2 genome from the cerebrospinal fluid (CSF) of patients with either CNS or PNS diseases. More recently, a systematic literature review on CSF testing in patients with COVID‐19 found that 17 out of 304 reviewed cases had a positive SARS‐CoV‐2 PCR in the CSF.^17^ However, a subsequent large multicenter study that tried to characterize the CSF profile of COVID‐19 patients with neurological involvement demonstrated negative SARS‐CoV‐2 CSF PCR in 76 out 76 samples.^18^ Overall, these data seem to suggest that direct invasion of the CSF is very unlikely, if occurring at all, and positive cases are more likely to represent contamination rather than a true infection. Tissue invasion can however occur albeit negative CSF studies. A meta‐analysis on brain autopsy findings from 20 papers and 184 COVID‐19 patients found evidence of SARS‐CoV‐2 brain invasion in 53.5% and 27.7% of cases by RT‐PCR and immunohistochemistry, respectively.^19^ Viral proteins have been found in cranial nerves originating from the lower brainstem and in isolated cells of the brainstem.^20^ Viral particles compatible with SARS‐CoV‐2 have been identified by electron microscopy in olfactory bulb and frontal lobe tissue.^21^, ^22^ The clinical significance of such findings remains unclear, as it does not correlate with neuropathological evidence of neuronal damage or neuroinflammation nor, importantly, with the occurrence of neurological symptoms.^20^ In a series of muscle and nerve specimens from 35 patients who died from COVID‐19, Suh et al did not find any evidence of viral invasion by SARS‐CoV‐2 nucleocapsid immunohistochemistry (IHC), although the authors speculate that viral RNA may have been cleared from muscle and nerve tissue before death, given the specimens were collected post‐mortem.^23^ In a series of post‐mortem diaphragm muscle specimens from 26 critically ill patients with COVID‐19, four patients (15.4%) had evidence of viral invasion by RT‐PCR, and the authors suggest that ACE‐2 expressed at the myofiber membrane could provide an entry point for SARS‐CoV‐2.^24^ In situ hybridization localized the RNA to inside the sarcolemma.^24^ These data suggest that although possible, the direct invasion mechanism is not a universal phenomenon, and its clinical significance remains unclear.

**TABLE 1:** Demographic, clinical, laboratory, electrophysiological, and imaging features of patients with GBS and COVID‐19 based on published literature

	ADIP	AMSAN	AMAN	Mixed	NA	All GBS
References	[36, 37, 38, 39, 40, 41, 42, 43, 44, 45, 46, 47, 48, 49, 50, 51, 52, 53, 54, 55, 56, 57, 58, 59, 60, 61, 62, 63, 64, 65, 66, 67, 68, 69, 70, 71, 72, 73, 74, 75]	[57, 67, 76, 77, 78, 79, 98]	[67, 80, 81, 82, 97]	[83, 84, 85]	[65, 71, 82, 85, 86, 87, 88, 89, 90, 91, 92, 93, 94, 95, 96]	[36, 37, 38, 39, 40, 41, 42, 43, 44, 45, 46, 47, 48, 49, 50, 51, 52, 53, 54, 55, 56, 57, 58, 59, 60, 61, 62, 63, 64, 65, 66, 67, 68, 69, 70, 71, 72, 73, 74, 75, 76, 77, 78, 79, 80, 81, 82, 83, 84, 85, 86, 87, 88, 89, 90, 91, 92, 93, 94, 95, 96, 97]
No. of patients % (n/total)	59.8 (55/92)	13.0 (12/92)	5.4 (5/92)	3.3 (3/92)	18.5 (17/92)	100 (92/92)
Type of study, no. of patients						
CR	31	5	4	2	10	52
CS	18	7	1	—	6	32
Other	6	—	—	1	1	8
Age of onset (mean ± SD, range)	58.1 ± 13.8 (11‐94)	58.6 ± 17.2 (23‐88)	33.4 ± 20.7 (15‐57)	45 ± 28.2 (21‐76)	50.9 ± 20.4 (18‐84)	55.2 ± 17.3 (11‐94)
Gender %males (n/total)	60 (33/55)	58.3 (7/12)	100 (5/5)	100 (3/3)	52.9 (9/17)	62.0% (57/92)
Onset relative to COVID mean ± SD days (range)	13.5 ± 8.7 (−8, 33)	9.6 ± 8.9 (−3, 27)	13.6 ± 12.8 (3, 18)	15.7 ± 5.5 (10, 21)	11.4 ± 6.3 (3, 21)	12.2 ± 8.3 (−8, 33)
GBS phenotype % (n)						
Classic	76.4 (42)	91.7 (11)	—	66.7 (2)	58.8 (10)	70.7 (65)
Variant	16.4 (9)	8.3 (1)	100 (5)	33.3 (1)	5.9 (1)	18.5 (17)
MFS	7.2 (4)	—	—	—	35.3 (6)	10.9 (10)
GBS presenting symptoms % (n)						
Motor LE	32.7 (18)	50 (6)	0	0	35.3 (6)	32.6 (30)
Motor UE	1.8 (1)	0	20.0 (1)	0	0	2.2 (2)
Tetraparesis	29.1 (16)	16.7 (2)	0	0	17.6 (3)	22.8 (21)
Sensory	60.0 (33)	33.3 (4)	0	100 (3)	47.1 (8)	52.2 (48)
Pain	5.5 (3)	0	40.0 (2)	0	11.8 (2)	7.6 (7)
Cranial nerve	10.9 (6)	16.7 (2)	0	0	16.7 (7)	16.3 (15)
Autonomic	0	0	20.0 (1)	0	0	1.1 (1)
Respiratory failure % (n)						
Yes	34.6 (18)	33.3 (4)	66.7 (2)	0	15.4 (2)	32.1 (26)
No	65.4 (34)	66.7 (7)	33.3 (1)	100 (2)	84.6 (11)	67.9 (55)
Autonomic involvement % (n)						
Yes	17.0 (8)	25.0 (2)	33.3 (1)	50.0 (1)	41.7 (2)	19.4 (14)
No	83.0 (39)	75.0 (6)	66.7 (2)	50.0 (1)	83.3 (10)	80.6 (58)
Time to nadir mean ± SD days (range)	6.2 ± 3.9 (2‐15)	5.8 ± 5.0 (2‐14)	3	3	7.7 ± 8.7 (1‐25)	6.1 ± 4.9 (1‐25)
CSF findings						
ACD % (n)	68.1 (32)	90.0 (9)	75 (3)	100 (3)	84.6 (11)	75.3 (58)
PCR +/n tested	0/29	0/6	0/4	0/1	0/7	0/47
Antiganglioside ab +/n tested	1/27	0/3	1/3	½	3/9	6/44
DDx labs +/n tested	0/23	0/2	0/2	½	0/2	1/31
MRI findings						
Brain (+, − )	2+, 11−	1+, 3−	0, 3−	1+, 0	2+, 5−	6+, 22−
Spine (+, − )	6+, 12−	1+, 4−	1+, 2−	0, 1−	0+, 4−	8+, 23−
NA	24	9	0	1	6	40
Brighton criteria % (n)						
I	78.4 (40)	72.7 (8)	50.0 (2)	100 (2)	7.1 (1)	64.6 (53)
II	21.6 (11)	27.3 (3)	50.0 (2)	0	64.3 (9)	30.5 (25)
III	0	0	0	0	28.6 (4)	4.9 (4)
GBS therapy % (n)						
IVIG	88.9 (48)	75.0 (9)	100 (5)	33.3 (1)	64.7 (11)	81.3 (74)
PEX	11.1 (6)	16.7 (2)	0	100 (3)	23.5 (4)	16.5 (15)
ICU	38.9 (21)	41.7 (5)	25.0 (1)	66.7 (2)	23.5 (4)	36.3 (33)
IV	22.2 (12)	25.0 (3)	25.0 (1)	33.3 (1)	17.6 (3)	21.9 (20)
NIV	1.9 (1)	8.3 (1)	0	0	0	2.2 (2)
Steroids	1.9 (1)	0	0	33.3 (1)	5.9 (1)	3.3 (3)
No treatment	1.9 (1)	0	0	0	11.8 (2)	3.3 (3)
GBS outcome (DS) % (n)						
≤2/6	59.0 (23)	33.3 (3)	40.0 (2)	100 (2)	62.5 (10)	55.5 (40)
3‐4/6	23.1 (9)	33.3 (3)	45.0 (2)	0	31.1 (5)	26.3 (19)
5‐6/6	17.9 (7)	33.3 (3)	20.0 (1)	0	6.3 (1)	16.7 (12)
Death	3.6 (2/55)	25.0 (3/12)	0	0	5.8 (1/17)	6.5 (6/92)

*Note*: Percentages are observed cases/cases where the information is available, unless otherwise reported.

Abbreviations: ACD, albuminocytologic dissociation; AIDP, acute inflammatory demyelinating polyneuropathy; AMSAN, acute motor sensory axonal neuropathy; Antiganglioside ab, antiganglioside antibodies; CR, case reports; CS, case series; DDx, differential diagnosis; DS, disability score; GBS, Guillain‐Barré syndrome; IV, invasive ventilation; IVIG, intravenous immunoglobulin; LE, lower extremity; NIV, non‐invasive ventilation; PEX, prefer plasmapheresis; UE, upper extremity; +/n tested, positive cases/overall tested cases.

The “post‐infectious” immune‐mediated hypothesis is supported by evidence that COVID‐19 causes a proinflammatory state due to the release of multiple cytokines, such as IL1, IL6, and TNF, as well as immune‐cell hyperactivation.^14^ The umbrella term “cytokine storm” has been used to describe this phenomenon, although its appropriateness for COVID‐19 is still debated.^25^ In the lung, this has been linked to the progression towards acute respiratory distress syndrome (ARDS).^25^ This mechanism has been proposed for other systemic complications of the disease, such as skin vasculitis, Kawasaki‐like syndrome, myocarditis, and hemophagocytic lymphohistiocytosis.^25^ Similar to other systems, vasculitis may affect cerebral small vessel causing stroke.^26^ In the PNS, individual reports of multiplex mononeuropathy suggested possible vasculitis that, however, could not be confirmed due to the lack of neuropathological data.^27^, ^28^, ^29^, ^30^

The isolation of pathogenic anti‐neuronal antibodies (eg, anti‐contactin‐associated protein 2),^31^ has been invoked as a proof of the immune‐mediated mechanism underlying post‐COVID‐19 myelitis and encephalitis.^14^ Although uncommon in our review (Table 1), the presence of disease‐specific antibodies, such as anti‐ganglioside antibodies has been observed in post‐COVID neuropathies. The shared pathogenetic hypothesis is that the molecular mimicry between SARS‐CoV‐2 surface proteins and self‐antigens may lead to the production of autoantibodies targeting neuronal antigens or nodal/paranodal proteins in the CNS and PNS, respectively. Although there is clinical evidence that some of the high‐affinity SARS‐CoV‐2‐neutralizing antibodies cross‐react with human self‐antigens, including self‐antigens found in the CNS, their ability to cross the brain‐ or nerve‐blood barrier has not been demonstrated.^32^ By using in silico analysis, Keddie et al demonstrated that there is no linear homology between SARS‐CoV‐2 proteins and any axonal or myelin surface proteins, thus, making the molecular mimicry hypothesis unlikely.^33^

An alternative immune‐mediated hypothesis has been proposed by Suh et al.^23^ The authors conducted a post‐mortem histopathological study on the psoas muscle and femoral nerve of 35 patients who died of severe COVID‐19 compared to 10 critically‐ill patients who were negative for SARS‐CoV‐2 but died during the COVID‐19 pandemic.^23^ They observed overexpression of the major histocompatibility complex‐1 (MHC‐1) in the muscle specimens of 25 out of 35 COVID‐19 patients as opposed to only one control; nine out of 35 nerve specimens showed evidence of inflammation as perivascular and/or endoneural inflammatory cells, whereas no evidence of neuritis was seen among controls. Finally, they observed abnormal expression of myxovirus resistance protein A (MxA) in the capillaries of nine muscles and seven nerve biopsies, as opposed to only one control muscle biopsy. The authors suggest that muscle and nerve damage may be secondary to the release of inflammatory cytokines, and more specifically of type 1 interferon, which is normally part of the protective response towards viral infection, but when overexpressed, can cause abnormal expression of MxA. The overexpression of this type 1 interferon‐induced protein has been observed in endothelial cells and surrounding dermal and epidermal tissues in dermatomyositis, systemic lupus erythematosus, and has been associated with some dermatologic complications of COVID‐19, such as chilblain‐like lesions.^34^ However, there are multiple limitations to this study, such as the inclusion of a selected group of critically ill patients, the role of comorbid diseases and medications, specifically COVID‐19 therapies (hydroxychloroquine, remdesevir, tocilizumab, immune checkpoint inhibitor), the lack of clinical data (such as at the time of collection; whether the psoas or the femoral nerve were clinically affected), of a neurologic exam, and limitations derived from the histological techniques used (specimens were fixed in formalin and no frozen nor plastic sections were available).

An additional effect of uncontrolled systemic inflammation is the occurrence of coagulopathy resulting mainly, although not exclusively, in venous thromboembolic events.^35^ In the CNS, this has been linked to an increased incidence of stroke in specific epidemiological scenarios, while its significance for PNS complications remains unclear. Some of the neuropathies secondary to COVID‐19 in our review could be secondary to thrombotic mechanisms, but much needed pathological data remain unavailable.

### 2.2 Guillain‐Barré syndrome

We identified 63 publications and 92 patients,^36^, ^37^, ^38^, ^39^, ^40^, ^41^, ^42^, ^43^, ^44^, ^45^, ^46^, ^47^, ^48^, ^49^, ^50^, ^51^, ^52^, ^53^, ^54^, ^55^, ^56^, ^57^, ^58^, ^59^, ^60^, ^61^, ^62^, ^63^, ^64^, ^65^, ^66^, ^67^, ^68^, ^69^, ^70^, ^71^, ^72^, ^73^, ^74^, ^75^, ^76^, ^77^, ^78^, ^79^, ^80^, ^81^, ^82^, ^83^, ^84^, ^85^, ^86^, ^87^, ^88^, ^89^, ^90^, ^91^, ^92^, ^93^, ^94^, ^95^, ^96^, ^97^, ^98^ including 51 case reports, 11 case series, and one single cross‐sectional study reporting GBS in concomitance or after COVID‐19. Interestingly, two cases clustered in the same family, either representing a chance finding, an expression of common antecedent COVID‐19, or unknown heritable factors.^85^ Table 1 summarizes our results.

**TABLE 2:** Summary of literature reports on isolated cranial neuropathies temporally associated with COVID‐19

Nerve	References	Age (years)	Sex	Temporal association (days)	Topography	Distinctive features	EDX	Imaging	CSF (proteins; cells; RT‐PCR)	Treatment	Outcome
**I**	[111]	21	M	0	B	None	NA	T2‐hyperintensity	NA	HXQ (COVID‐19)	Persistent anosmia
	[112]	70	M	NA	B	Leukocytic infiltrates and axonal damage on autopsy	NA	NA	NA	HXQ (COVID‐19)	NA
	[112]	79	M	NA	NA	Leukocytic infiltrates and axonal damage on autopsy	NA	NA	NA	HXQ (COVID‐19)	NA
**II**	[113]	62	F	+21	L	Non convulsive status epilepticus	Delayed VEP	Enhancement of nerve and cortex	↑; N; Neg	Tocilizumab, DEXA (COVID‐19)	Persistent nerve enhancement
	[114]	50	F	+2	R	Pain; uveitis and papillitis	NA	NA	N; N; Neg	Oral and topical steroids	Macular atrophy
**III**	[115]	24	F	+3	R	None	NA	NA	NA	Chloroquine, azithromycin (COVID‐19)	Complete
	[116]	62	M	0	L	None	NA	NA	NA	Antiviral, IVIG, IV steroids	Death due to respiratory failure
**VI**	[96]	71	F	NA	R	None	NA	Nerve enhancement	N; N; NA	HXQ (COVID‐19)	No change
	[93]	39	M	+3	B	Possible MFS	NA	NA	↑; N; Neg	Supportive	Complete
	[135]	52	M	+2	L	None	NA	NA	NA	Supportive	Complete
	[135]	43	F	+3	L	None	NA	NA	NA	Supportive	NA
**VII**	[117]	35	F	+2	R	Unilateral ageusia	NA	NA	N; N; Neg	Phytotherapy	Complete
	[118]	37	M	+12	R	Myocarditis; anti‐ganglioside abs	NA	NA	↑; ↑; Neg	Doxycycline	Complete
	[119]	27	M	+6	L	Severe headache	NA	Nerve enhancement	N; N; Neg	Oral steroids, valacyclovir	No change
	[120]	57	M	+7	L	NA	Absent BR	NA	N; N; Neg	Supportive	Complete
	[121]	43	F	0	R	NA	NA	NA	NA	Oral steroids	Partial
	[121]	25	F	0	R	NA	NA	NA	N; N; Neg	Oral steroids, acyclovir	Complete
	[121]	33	F	0	R	NA	NA	NA	NA	Oral steroids, acyclovir	Partial
	[121]	26	F	+2 to +10	R	NA	NA	Nerve enhancement	N; N; Neg	Oral steroids	Complete
	[121]	50	F	+2 to +10	L	NA	NA	NA	↑; N; Neg	Oral steroids	Partial
	[121]	38	F	+2 to +10	L	NA	NA	NA	N; N; Neg	Supportive	Complete
	[121]	39	F	+2 to +10	R	NA	NA	NA	N; N; Neg	Oral steroids	Complete
	[121]	34	M	+2 to +10	L	NA	NA	NA	N; N; Neg	IV steroids	Complete
	[122]	48	M	+8	L	None	NA	NA	NA	IV steroids	Partial
	[123]	25	M	0	L	NA	NA	NA	NA	Oral steroids, valaciclovir	Partial
	[123]	34	M	+8	R	NA	NA	NA	NA	Oral steroids, valaciclovir	Complete
	[124]	28	F	+3	R	36 weeks of gestation	NA	NA	NA	Oral steroids, valacyclovir	Complete
	[125]	20	M	+7	B	EBV co‐infection	Delayed BR; denervation	Bilateral nerve enhancement	↑; N; Neg	NA	Complete
	[126]	44	M	NA	B	Severe ageusia	NA	NA	↑; N; Neg	IVIG	Partial
	[127]	61	M	+10	B	NA		NA	↑; N; Neg	Oral steroids	No change
**VII+V**	[128]	58	M	+5	L	Dysgeusia	NA	NA	NA	Valacyclovir	Complete
**VIII**	[129]	52	M	+3	L	Preceded by tinnitus	NA	NA	NA	Intratympanic and IV steroids	Complete
	[130]	29	M	NA	R	Asymptomatic COVID‐19 testing	NA	NA	NA	HXQ (COVID‐19)	Complete
	[131]	60	M	+12	B	Concomitant delirium; tinnitus	Altered BAER	Enhancement R cochlea and temporal bone	NA	Intratympanic steroids	NA
**X + XII**	[132]	62	M	+16	L	Prone ventilation	NA	NA	NA	Supportive	No change
**XII**	[133]	42	M	+30	B	CBH; delirium; prone ventilation	Denervation	NA	N; N; Neg	IVIG	Partial
	[134]	24	M	+13	R	Delirium; orothracheal intubation	NA	NA	NA	Physical therapy	Partial

Abbreviations: B, bilateral; BAER, brainstem auditory evoked response; BR, blink reflex; CBH, Claude Bernard Horner syndrome; CIPN, critical illness polyneuropathy; DEXA, dexamethasone; HXQ, hydroxychloroquine; L, left; N, normal; NA, not available; Neg, negative; R, right; VEP, visual evoked potentials.

Three cohorts of consecutive COVID‐19 patients presenting with GBS have been published in Italy (30 cases),^99^ Spain (11 cases),^100^ and UK (25 cases, including 13 “definite” and 12 “probable COVID‐19”).^33^ We did not include these additional 66 cases in Table 1 as the clinical information were not available for individual cases. These cohorts were considered for comparison purposes.

All continents except Australia were represented, with the majority of publications coming from Europe (37 out of 63) and remaining from Asia (13), North America (10), Africa (two) and South America (one). Strikingly, some significant COVID‐19 hotspots, such as South America, were under‐represented possibly as an effect of publication bias. Large global surveillance studies will be necessary to understand whether the epidemiology of GBS related to COVID‐19 has distinctive features, including geographical clustering, as seen during the Zika pandemic.^101^

The clinical features of COVID‐19 are detailed in Table 3. The majority of patients (75 out of 92) were diagnosed by positive RT‐PCR testing on nasopharyngeal swab, with only a few diagnoses (13 out of 92) relying on positive serology (IgG and/or IgM). Fever and cough were the two most common presenting symptoms (64.3% and 66.7%, respectively), with only few (3.4%) patients being asymptomatic on presentation. Approximately 9% of patients developed ARDS requiring invasive ventilation. Increased inflammatory markers and/or lymphocytopenia were the most common laboratory findings (96.9% of cases, 62 out of 64 patients where this information was available). Chest imaging showed ground‐glass opacities (GGO) in 75% of cases (48 out of 64). Therapeutic choices reflected initial uncertainties regarding the most effective regimen, with hydroxychloroquine being the preferred medication in 43.4% of treated patients followed by antivirals other than remdesevir in 32.5% cases. The overall outcome of the respiratory disease was positive with the majority of patients (75.6%) being asymptomatic upon discharge. In only two cases (3.4%), a fatal outcome was attributed to COVID‐19 and respiratory‐related complications.

**TABLE 3:** Clinical, laboratory, and imaging features of COVID‐19 in patients with GBS

	ADIP	AMSAN	AMAN	Mixed	NA	All GBS
References	[36, 37, 38, 39, 40, 41, 42, 43, 44, 45, 46, 47, 48, 49, 50, 51, 52, 53, 54, 55, 56, 57, 58, 59, 60, 61, 62, 63, 64, 65, 66, 67, 68, 69, 70, 71, 72, 73, 74, 75]	[57, 67, 76, 77, 78, 79, 98]	[67, 80, 81, 82, 97]	[83, 84, 85]	[65, 71, 82, 85, 86, 87, 88, 89, 90, 91, 92, 93, 94, 95, 96]	[36, 37, 38, 39, 40, 41, 42, 43, 44, 45, 46, 47, 48, 49, 50, 51, 52, 53, 54, 55, 56, 57, 58, 59, 60, 61, 62, 63, 64, 65, 66, 67, 68, 69, 70, 71, 72, 73, 74, 75, 76, 77, 78, 79, 80, 81, 82, 83, 84, 85, 86, 87, 88, 89, 90, 91, 92, 93, 94, 95, 96, 97]
No. of patients % (n/total)	59.8 (55/92)	13.0 (12/92)	5.4 (5/92)	3.3 (3/92)	18.5 (17/92)	100 (92/92)
COVID‐19 RT PCR % (n)						
Positive	81.8 (45)	90.9 (10)	100.0 (4)	100.0 (2)	100.0 (14)	88.2 (75)
Negative	18.2 (10)	9.1 (1)	0	0	0	11.8 (11)
COVID‐19 serology % (n)						
+IGG	16.4 (9)	16.7 (2)	0	0	0	12 (11)
+IGM	0	0	0	0	0	0
+IGG & +IGM	1.8 (1)	0	20 (1)	0	0	2.2 (2)
−IGG or −IGM	3.6 (2) (IgM)	0	0	0	0	2.2 (2)
NA	81.8 (45)	83.3 (10)	80 (4)	100 (3)	100 (17)	85.9 (79)
Chest imaging % (n)						
Positive	60 (33)	75 (9)	20 (1)	33.3 (1)	23.5 (4)	52.2 (48)
Negative	16.4 (9)	16.7 (2)	20 (1)	33.3 (1)	11.8 (2)	16.3 (15)
NA	23.6 (13)	8.3 (1)	60 (3)	33.3 (1)	64.7 (11)	31.5 (29)
COVID‐19 symptoms % (n)						
Fever	58.5 (31)	63.6.0 (7)	80.0 (4)	66.7 (2)	75.0 (12)	64.3 (56)
Dyspnea	22.6 (12)	45.5 (5)	20.0 (1)	66.7 (2)	12.5 (2)	25.3 (22)
Cough	67.9 (36)	81.8 (9)	40.0 (2)	66.7 (2)	56.3 (9)	66.7 (58)+
Headache	17.0 (9)	0	20.0 (1)	33.3 (1)	6.3 (1)	13.8 (12)
Other UR symptoms	13.2 (7)	9.1 (1)	(1)	0	18.8 (3)	13.8 (12)
Myalgia	17.0 (9)	9.1 (1)	0	0	18.8 (3)	14.9 (13)
Anosmia and/or ageusia	32.1 (17)	18.2 (2)	0	0	25.0 (4)	26.4 (23)
GI	20.8 (11)	0	20.0 (1)	33.3 (1)	25.0 (4)	19.5 (17)
Other symptoms	7.5 (4)	0	0	0	0	4.6 (4)
Asymptomatic	5.7 (3)	0	0	0	0	3.4 (3)
COVID‐19 labs % (n)						
Inflammatory markers	62.5 (25)	77.7 (7)	50.0 (1)	0	45.5 (5)	59.4 (38)
Lymphocytopenia	32.5 (13)	66.7 (6)	50.0 (1)	50.0 (1)	217.3 (3)	37.5 (24)
Normal	20.0 (8)	0	50.0 (1)	50.0 (1)	45.5 (5)	23.4 (15)
COVID‐19 ARDS % (n)						
Yes	11.1 (6)	18.2 (2)	0	0	5.9 (1)	9.1 (9)
No	88.9 (48)	81.8 (9)	100 (4)	100 (3)	94.1 (16)	90.9 (80)
COVID‐19 therapy % (n)						
Steroids	14.3 (7)	16.7 (2)	25.0 (1)	0	6.7 (1)	13.2 (11)
Remdesevir	0	16.7 (2)	0	0	0	2.4 (2)
Other antivirals	36.7 (18)	50.0 (6)	0	0	20.0 (3)	32.5 (27)
Hydroxicloriquine	40.8 (20)	58.3 (7)	25.0 (1)	33.3 (1)	46.7 (7)	43.4 (36)
Antibiotics	16.3 (8)	33.3 (4)	50.0 (2)	0	13.3 (2)	19.3 (16)
None	46.9 (23)	16.7 (2)	25.0 (2)	66.7 (2)	33.3 (5)	40.9 (34)
COVID‐19 outcome % (n)						
Symptomatic	24.2 (8)	14.3 (1)	250. (1)	66.7 (2)	0	20.7 (12)
Asymptomatic	72.7 (24)	71.4 (5)	75.0 (3)	33.3 (1)	100 (11)	75.6 (44)
Death	3.0 (1)	14.3 (1)	0	0	0	3.4 (2)

*Note*: Percentages are observed cases/cases where the information is available, unless otherwise reported.

Abbreviations: AIDP, acute inflammatory demyelinating polyneuropathy; AMSAN, acute motor sensory axonal neuropathy; GBS, Guillain‐Barré syndrome; GI, gastrointestinal; UR, upper respiratory.

The clinical features of GBS patients with COVID‐19 are detailed in Table 1. The mean age of onset was 55.2 ± 17.3 years (median 58, range 11‐94 years) with a bimodal age distribution showing the highest peak in the 50 to 75 age group (61 out of 92, 66.3%) and a second lower peak in the 15–35 age group (12 out of 92, 13%). These figures are comparable to meta‐analysis on the broader GBS population^102^ and to the three COVID‐19/GBS cohorts mentioned above.^33^, ^99^, ^100^

Among COVID‐19/GBS cases, there was a male preponderance with an M:F ratio of 1.6 (57:35), within the range reported for non‐COVID‐19 GBS (1.78, 95% CI 1.36‐2.33),^103^ but lower than what reported in the Italian and UK cohorts (2.75 and 4.0, respectively).^33^, ^99^

In all except three cases,^37^, ^71^, ^79^ the neurologic manifestations followed COVID‐19 symptoms or diagnosis, with a median time interval of 13 days (mean 12.2 ± 8.3 days, range from 8 days before to 33 days after COVID‐19), similar to the findings from the UK and Spanish cohort (median 12 and 10 days, respectively),^33^, ^100^ but strikingly lower than the Italian cohort (median 23 days).^99^ Overall, this time lag suggests a post‐infectious process, and is similar to what reported in up to 70% of post‐infectious GBS cases in pre‐COVID era.^104^, ^105^ More specifically, this temporal pattern is similar to GBS cases following various viral infections (ie, EBV, CMV, HEV, and influenza A), but different from Zika‐related GBS, where neurological symptoms occur after a shorter time interval with a median of 7 days.^101^

The diagnosis of GBS was based on the Brighton criteria, with all except four cases reaching level 1 or 2 of diagnostic certainty. All GBS phenotypes were represented, with 70.7% of patients (65 cases) presenting with a classic sensorimotor onset. Miller‐Fisher syndrome (MFS), including incomplete subtypes, represented 10.9% of cases (10 patients). GBS variants were diagnosed in 18.4% of cases (17 patients). Among them, the most common was pure motor GBS (eight cases) and bifacial weakness with paresthesia (four cases). The most common presenting symptom was lower limb weakness (57.6%, 53 out of 92 cases), followed by sensory symptoms (52.2%; 48 patients including two cases with sensory ataxia). Cranial nerve involvement was described in 16.3% of cases (15 patients). Overall, these clinical features are similar to what seen for GBS in the general population^106^ and in the COVID‐19 cohorts.^33^, ^99^, ^100^ Few patients had an onset with atypical clinical features such as: unilateral facial palsy,^68^, ^71^ involvement of the vestibulocochlear cranial nerves (clinically and on neurophysiological and imaging studies),^84^ dysautonomia preceding motor symptoms,^97^ and syndrome of inappropriate antidiuretic hormone secretion (SIADH).^49^, ^88^

Respiratory failure related to neuromuscular weakness rather than COVID‐19 occurred in 32.1% of patients reported in Table 1 **(**26 cases), whereas 21.7% of patients required invasive ventilation (20 patients). These figures are comparable to the 25% reported in the literature for GBS in the general population,^104^ and in the COVID‐19/GBS cohorts (range 17%‐28%).^33^, ^99^, ^100^ Autonomic dysfunction occurred in 19.4% of GBS cases (14 cases), but diagnostic methods and severity of dysautonomia were largely variable among studies. This variability has been observed in larger population studies for non‐COVID‐19 GBS as well.^104^ Filosto et al found higher rates of hypotension in their COVID‐19/GBS cohort when compared to a control GBS cohort, but this finding may likely reflect a higher proportion of critically ill patients requiring intensive care unit (ICU) admission (50% vs 17.6%).^99^ In the Spanish COVID‐19/GBS cohort^100^ the ICU admission rate was 36.4%, which is similar to the 36.7% we found among cases reviewed in Table 1.

The most common electrophysiological diagnosis was AIDP (59.8%, or 55 out of 92 total cases) with axonal variants (AMAN and AMSAN) being reported in 18.5% cases (17 out of 92). This distribution reflects the epidemiology of non‐COVID‐19 GBS, where axonal variants are relatively uncommon in Europe and North America, which contributed to the majority of cases in our review. Among the 14 patients from Asia or South America and for whom an electrophysiological diagnosis was reported, eight (57.1%) were diagnosed with an axonal variant, within the 30% to 65% range reported in non‐COVID‐19 populations.^104^ In the UK and Italian COVID‐19 cohorts, a higher than expected frequency of AIDP was noted,^33^, ^99^ although it was statistically significant only in one study.^99^ This likely reflected a higher than expected prevalence of axonal forms in their GBS control cases (up to 41.2%; seven out of 17 cases).^99^

In a separate study on the same Italian COVID‐19 cohort, the electrophysiological features of AIDP were compared to non‐COVID‐19 AIDP.^107^ Distinctive features among COVID‐19 patients were higher percentage of cases with absent F waves, which was attributed to motor neuron hypoexcitability, and increased duration of distal compound motor action potential (CMAP) without changes in distal latencies, which was interpreted as conduction slowing within muscle fibers. A major confounder in this study was that a large proportion of COVID‐19 AIDP patients had a critical illness, which in some of the cases was due to the underlying lung infection.^107^

Laboratory testing disclosed albumin‐cytological dissociation in 75.3% of cases, comparable to the 64% seen in the overall GBS population,^104^ with some variability in possible relation to the timing of CSF assay and clinical onset. This finding again supports a classical post‐infectious pathophysiology. No study reported CSF cell counts above 50 cells, and none of the 47 CSF PCR studies tested positive for SARS‐Cov‐2 genome, except for one patient in the Spanish cohort.^100^ To date, the latter remains an isolated finding among published literature.

Testing for autoantibodies was performed in 44 patients and it was positive in six, including two patients with anti‐GM1 (one AMAN and one mixed electrophysiology),^81^, ^95^ one classic GBS with anti‐GM2^92^ and another with anti‐GD1a.^108^ Finally, one patient with classic presentation tested positive for anti‐pan‐neurofascin IgM^84^ and had a severe course with early cranial and respiratory involvement. Unexpectedly, among the 11 MFS cases only one tested positive for anti‐GD1b^93^ and none for anti‐GQ1b, as compared to 90% positivity in non‐COVID‐19 MFS patients.^104^ The same finding was reported in the UK cohort.^33^

Brain and spine MRI were performed in 28 and 31 cases. Common findings, although present in less than 30% of cases, included enhancement of cranial nerves, spinal nerve roots, and cauda equina. Far from being specific to COVID‐19, these findings added diagnostic certainty to GBS diagnosis.

The initial concern that intravenous immunoglobulin (IVIG) might impair the humoral immunity towards SARS‐CoV‐2 prompted some clinicians to prefer plasmapheresis (PEX) as first‐line therapy. However, there was no report of clinical deterioration after IVIG, which was the preferred treatment in 81.3% of cases (74 patients). This was the same approach reported in the GBS/COVID‐19 cohorts.^33^, ^99^, ^100^ The data on clinical outcomes were scarce among published cases. For example, Filosto et al based the good response to treatment in 85% of cases on the clinical impression only, which was comparable to non‐COVID‐19 GBS cases (ie, 94%).^99^

GBS disability scores upon discharge are reported in Table 1. Not all the studies reported this score, and when possible, we reconstructed the outcomes based on the clinical descriptions. Overall, 43.1% (19 patients) had poor outcome (GBS disability score [DS] ≥ 3). In the UK and Italian cohort, between 40% and 50% of COVID‐19 GBS cases had a poor outcome, similar to what found in a non‐COVID‐19 population used as control.^33^, ^99^ However, these percentages are higher than the 20% reported in the literature for the general GBS population.^104^, ^108^ The mortality rate among COVID‐19/GBS cases was 6.5% (Table 1), similar to reports in the general population.^105^ No deaths related to GBS and neuromuscular weakness were reported in the three cohort studies.^33^, ^99^, ^100^

Overall, based on our extensive review of published literature, GBS phenotype among COVID‐19 patients did not show distinctive features. This conclusion was also reached by the UK cohort study.^33^ Few clinical findings, such as frequent need for ICU stay^99^, ^100^ and invasive ventilation,^33^ and possibly more severe disability outcomes (Table 1), could suggest that COVID‐19 may be a negative prognostic factor for GBS.

Whether Sars‐CoV‐2 may be an etiologic agent/trigger remains to be determined. The three large studies that were designed to address this specific question reached contradictory conclusions. In UK,^33^ GBS incidence was reported to have fallen during the pandemic, possibly because reduced social contacts and/or increased hand hygiene had decreased the circulation of other etiologic agents, such as C. Jejuni or respiratory viruses. The same finding was reported in Singapore.^109^ The Spanish study involved 61 emergency departments during the first 2 months of the pandemic and showed that GBS incidence was 0.15% in patients with COVID‐19 and 0.02% in those without.^100^ This apparent excess of GBS among COVID‐19 cases was due to a decrease of incidence among the non‐COVID‐19 population, confirming the trend showed in UK and Singapore. Indeed, the number of GBS cases recorded in March to April 2020 was the same of that in the same months of 2019 (23 vs 21).^100^ In Northern Italy, between March and April 2020, a 2.6‐fold increase in the incidence of GBS with 3.3‐fold decrease in non‐COVID‐19 cases was reported.^99^ However, this finding should be interpreted with caution because there was an overlap in the confidence intervals between the two incidences due to the small number of non‐COVID‐19 GBS cases and the short period of observation. Similarly, one retrospective study^108^ from another region in North‐Eastern Italy reported 3.5 cases/month in March to April 2020 compared to the expected rate of 0.67 cases/month that reflected an increase from four to eight GBS cases in 6 months. However, none of the patients tested positive for Sars‐Cov‐2 at the nasopharyngeal swab, and only one was reported with positive serum and CSF serology, thus, arguing against an etiological correlation.

Larger and longer case‐control studies and surveillance data from multiple geographic regions will ultimately be able to prove any epidemiological association between COVID‐19 and GBS. In this regard, a recent international prospective cohort study by the International GBS Outcome Study consortium enrolled incident GBS cases between 30 January 2020 and 30 May 2020 and found no increase in patient recruitment during the pandemic.^110^ A higher prevalence of COVID‐19 was noted among GBS cases when compared to the general population, but this could have been secondary to substantial recruitment bias.^110^

### 2.3 Cranial neuropathies

Isolated or multiple cranial neuropathies not associated with polyneuropathy or other neurological disorders have been reported (Table 2).^93^, ^96^, ^111^, ^112^, ^113^, ^114^, ^115^, ^116^, ^117^, ^118^, ^119^, ^120^, ^121^, ^122^, ^123^, ^124^, ^125^, ^126^, ^127^, ^128^, ^129^, ^130^, ^131^, ^132^, ^133^, ^134^ These included suspected bilateral olfactory neuropathy,^111^, ^112^ optic neuropathy,^113^, ^114^ oculomotor neuropathy either isolated^96^, ^115^, ^116^, ^135^ or associated with multiple cranial neuropathies,^93^, ^128^ unilateral^117^, ^118^, ^119^, ^120^, ^121^, ^122^, ^123^, ^124^ or bilateral facial nerve palsy,^125^, ^126^, ^127^ sensorineural hearing loss,^129^, ^130^, ^131^ and lower cranial nerve impairment.^132^, ^133^, ^134^ Facial nerve palsy was the most commonly reported with 20 cases, followed by isolated oculomotor nerve neuropathy. With few exceptions,^114^, ^135^ all cases were painless.

Olfactory dysfunction (ie, anosmia or hyposmia) is an early and frequent symptom of COVID‐19, reported by up to 80% of patients within the first 5 days of the disease.^136^, ^137^ One hypothesis is that Sars‐CoV‐2, similar to Sars‐CoV, can invade the olfactory bulb causing neuronal death or dysfunction. Few reports have shown isolated involvement of the olfactory bulbs by magnetic resonance imaging (MRI) contrast enhancement,^111^ T2 MRI hyperintensity,^138^ or leukocytic infiltrate and axonal damage on brain autopsy.^112^ Evidence that these symptoms may be transitory in the majority of patients has suggested a competitive mechanism on the olfactory receptors rather than a permanent cell damage.^137^ For yet unknown reasons, olfactory symptoms are strongly associated with gustatory dysfunction (ie, ageusia or dysgeusia).^136^

We identified two literature reports of isolated optic neuropathy.^113^, ^114^ An inflammatory mechanism was hypothesized based on MRI contrast enhancement in one case and evidence of papillitis and uveitis in the other one. The large ALBACOVID cohort in Spain reported a clinical diagnosis of optic neuropathy in 1 out of 841 patients.^2^ Few studies have demonstrated retinal abnormalities. Optical coherence tomography (OCT) changes have been described in a series of 11 COVID‐19^139^ but, interestingly, without any clinical correlate. Other subtle retinal abnormalities have been described, including larger retinal vein diameters and vascular lesions.^140^, ^141^, ^142^

The facial nerve has attracted much interest given the frequent occurrence of gustatory symptoms among COVID‐19 patients. Reports of severe unilateral dysgeusia leading to the diagnosis of ipsilateral facial palsy however remain isolated.^68^, ^117^, ^128^ A retrospective study conducted during the first wave of COVID‐19 in a single town in Northern Italy reported an increased incidence of Bell's palsy compared to the same period of 2019 (7.1 vs 4.1 cases per 100 000 inhabitants, RR 1.73).^143^ The clinical phenotypes were largely comparable between the two periods and no differences were reported in the response rates to steroid treatment. A key limitation was that the majority of patients with COVID‐19 were diagnosed clinically and not based on molecular testing. Another study found an unusually large cluster of six cases of Bell's palsy in a pediatric population, but the association with COVID‐19 was only clinically presumed as all cases either tested negative or were not tested at all for Sars‐CoV‐2.^144^ A prospective study conducted in Turkey reported a higher than expected seroprevalence of COVID‐19 (i.e. IgM and/or IgG positivity) among otherwise asymptomatic patients presenting with isolated unilateral facial palsy.^145^

Auditory complications have been described in COVID‐19 patients, with reports of sudden onset of unilateral^129^, ^130^ or bilateral^131^ sensorineural hearing loss. In one case,^131^ a likely inflammatory pathophysiology was suggested by MRI contrast enhancement of the ipsilateral cochlea and temporal bone. These findings were not confirmed by another MRI study.^129^ A larger survey among severe COVID‐19 survivors found self‐reported change in hearing and/or tinnitus in 16 out of 138 adults (13.2%) at 8 weeks after hospital discharge.^146^

Three case of hypoglossal nerve neuropathy have been reported.^132^, ^133^, ^134^ All occurred unilaterally and were temporally related with either endotracheal intubation or prone positioning, suggesting a iatrogenic etiology.

Based on available evidence, any direct etiological association between COVID‐19 and cranial neuropathies seems inconclusive. Larger and longer case‐control studies will be needed to address any causal link.

### 2.4 Chronic inflammatory demyelinating polyneuropathy and other neuropathies

Because GBS patients reported in Table 1 were not prospectively followed up, it is unclear whether some of them were in fact acute‐onset chronic inflammatory demyelinating polyneuropathy (CIDP), which is reported in up to 5% to 16% of patients in pre‐COVID‐19 studies.^147^

In our literature review, we did not find any report linking COVID‐19 to a new diagnosis of CIDP.

The potential of COVID‐19 to precipitate CIDP has been proposed in few reports. One case reported a 69‐year‐old man with a 6‐year history of CIDP on multiple immunosuppressive and immunomodulatory treatments who developed a clinical exacerbation in concomitance with COVID‐19.^148^ This presentation was unusual for this patient due to a more severe and extended phenotype that included respiratory failure, tetraparesis and cranial nerve involvement. The authors noted serum IL‐6 elevation and wondered if this could be a mechanism involved in CIDP exacerbation. The outcome was positive with almost complete clinical and electrophysiological recovery after two cycles of IVIG. A single additional report^149^ similarly described a more severe clinical picture after COVID‐19 with need for mechanical ventilation, however with relatively good prognosis after IVIG.

The above‐mentioned case series on femoral nerve biopsies of 35 COVID‐19 patients showed evidence of neuritis in 9 cases, of whom 4 also had myositis.^23^ The main histological findings were perivascular inflammation in 6 patients, endoneural infiltrates in 1, and both perivascular and endoneural inflammatory cells in 2. Cell infiltrates were mainly CD68‐positive histiocytes. None had signs or symptoms of GBS. However, no clinical data supported these findings, and some of the neuritis cases had comorbid conditions (diabetes) and/or had received treatments (such as pembrolizumab) that could have caused this presentation.^23^

Although COVID‐19 has been linked to histologically confirmed cutaneous vasculitis, Kawasaki‐like vasculitis and possibly CNS vasculitis,^150^ there are few evidences linking COVID‐19 to vasculitic neuropathies. An unusually large cluster of 11 multiplex mononeuritis among 69 patients admitted for severe COVID‐19 has been reported.^151^ None of these patients showed clinical or electrophysiological evidence of critical illness neuropathy/myopathy, and no focal demyelination at entrapment sites was noted to point towards prone positioning as a likely culprit. Electrodiagnostic studies demonstrated axonal loss in the affected nerves. The authors found their data to be consistent with a vasculitic pathogenesis, although no nerve pathology was performed to confirm this hypothesis. Asymmetric sensory‐motor polyneuropathy has been reported in some COVID‐19 patients.^152^, ^153^ Given the association with necrotic skin lesions in one of these cases, vasculitis was considered to be the most likely etiology.^152^ Two cohort studies reported the occurrence of subacute polyneuropathy temporally related to COVID‐19 that did not fulfill the diagnostic criteria for GBS.^4^, ^6^ However, no additional clinical and electrodiagnostic data were provided to make a specific diagnosis. Histological studies would be necessary to confirm this association, and more important to distinguish between true vasculitis and vasculitis mimics—that is, vascular thrombotic disease—whose occurrence may be more frequent in the setting of COVID‐19 induced hypercoagulability.

Reports of brachial plexopathy following COVID‐19 and unrelated to ICU stay or prone positioning are relatively scarce. One case of painful brachial plexopathy occurring about 3 weeks after COVID‐19 has been reported.^154^ This patient developed a purpuric rash on the ipsilateral hand and forearm which was deemed to be induced by thrombotic microvascular injury although no skin biopsy was performed. The authors argued that the peculiar pattern of axonotomy with sparing of some fascicles and severe denervation of other fascicles within the same trunks and cords along with the dermatologic findings suggested a thrombotic mechanism in the setting of COVID‐19‐induced hypercoagulability. A picture more consistent with neuralgic amyotrophy has been reported in four cases to date.^27^, ^28^, ^29^, ^30^ Typical symptoms of pain and shoulder or hand weakness started between one to several weeks after COVID‐19 onset. MRI findings included T2‐hyperintensity of ipsilateral cervical roots^30^ and increased T2 signal of affected muscles likely secondary to denervation‐related edema,^27^, ^28^ as would be expected for classic neuralgic amyotrophy. In addition, all cases were moderately responsive to high‐dose oral steroids. Peculiar features reported in individual cases were bilateral brachial plexus involvement,^27^ sparing of motor fibers^29^ and association with a systemic immune‐mediated process similar to multisystem inflammatory syndrome.^28^

The potential of COVID‐19 to cause small fiber neuropathy (SFN) has been postulated based on the occurrence of autonomic dysfunction among COVID‐19 patients presenting with GBS (Table 1). In one of such cases, autonomic dysfunction in the form of profuse sweating, constipation and erectile dysfunction preceded motor symptoms.^97^ In the ALBACOVID registry, 2.5% of patients were diagnosed with autonomic dysfunction.^2^ Although growing literature interest is directed toward SFN in “long COVID” (see below), in our review we did not find any additional literature report on isolated SFN being diagnosed in the acute setting.

### 2.5 Muscle and neuromuscular junction

Myalgia and asymptomatic CK elevation are common findings among COVID‐19 patients but do not correlate with clinical, electrodiagnostic, or histologic evidence of muscle damage nor with the severity of the underlying infection, and do not predict the subsequent development of myopathy.^2^

Rhabdomyolysis however may be more common in patients with severe COVID‐19; it may be a presenting symptom predicting worse outcomes.^155^, ^156^ Patients present with significant CK elevation, up to 33 000 UI in one study, and a spectrum of clinical findings, including myoglobinuria, acute kidney injury, weakness, which is proximal, lower limb‐dominant, acute and symmetric and neurogenic respiratory failure requiring mechanical ventilation.

A clinical and laboratory diagnosis of myopathy has been reported in 0.5% to 3.1% of patients with COVID‐19 depending on the study cohort.^2^, ^8^ However, no electrodiagnostic or biopsy studies were available for the majority of patients. In the ALBACOVID cohort, the occurrence of myopathy was predicted by longer ICU stay.^2^

**TABLE 4:** Literature review on COVID‐19‐induced myositis

Phenotype	References	Age (years)	Sex	Temporal association (days)	CK (initial)	Diagnosis	Treatment	Outcome
Proximal myopathy	[157]	38	M	+4	21 000	Clinical, lab	RRT, hydration	Complete recovery
Proximal myopathy	[158]	NA	NA	‐4	25 384	MRI, lab	Hydration	Prolonged ICU
Proximal myopathy	[159]	38	M	+3	42 670	Clinical, lab	Hydration	Complete recovery
Proximal myopathy	[10]	38	M	0	29 800	Clinical, lab, biopsy	Oral and IV steroids	Partial recovery
Proximal myopathy	[164]	40	M	+14	850	Clinical, EMG, MRI, biopsy	COVID‐19 treatment	Prolonged rehabilitation
Bulbar involvement	[160]	58	F	+21	700	Biopsy, MRI, EMG, lab (anti SSA, anti‐SAE‐1ANA), EM	IV steroids	Partial recovery, PEG
Dermatomyositis	[161]	64	M	Preceded COVID‐19	990	Clinical, lab (ANA)	IVIG, mycophenolate, oral steroids	Partial recovery
Dermatomyositis	[161]	50	F	Preceded COVID‐19	150	Clinical, lab (anti‐MDA5, SAE‐1)	IV steroids, MTX, cyclophosphamide	Death
Dermatomyositis	[161]	26	F	Preceded COVID‐19	8349	Clinical, lab (Mi2)	MTX, oral steroid, HXQ	Complete recovery
Dermatomyositis	[161]	46	M	Preceded COVID‐19	570	Clinical, lab (anti SAE)	HXQ, mycophenolate, MTX	Complete recovery
Paraspinal myositis	[162]	33	F	Preceded COVID‐19	NA	Spine (T/L) MRI	NA	Complete recovery
Paraspinal myositis	[162]	60	M	Preceded COVID‐19	NA	Spine (C/L) MRI	NA	Complete recovery
Paraspinal myositis	[162]	63	M	Preceded COVID‐19	NA	Spine (T/L) MRI	Intubation	Ventilator dependence
Paraspinal myositis	[162]	87	M	Preceded COVID‐19	NA	Spine (T/L) MRI	NA	Complete recovery
Paraspinal myositis	[162]	54	F	Preceded COVID‐19	NA	Spine (L) MRI	Intubation	Partial recovery
Paraspinal myositis	[162]	62	M	Preceded COVID‐19	NA	Spine (C/T/L) MRI	Intubation	Partial recovery
Paraspinal myositis	[162]	56	M	Preceded COVID‐19	NA	Spine (C/T/L) MRI	Intubation	Partial recovery
Muscle ischemia (bilateral thighs)	[163]	33	M	0	Elevated (not reported)	Clinical, lab, CT	Anticoagulation, fasciotomy, bilateral amputation	Lower limbs amputation

Abbreviations: EM, electronic microscopy; HXQ, hydroxychloroquine; MTX, methotrexate; NA, not available; RRT, renal replacement therapy; lab = CK elevation, or additional studies when indicated.

Growing literature has reported the occurrence of myositis in the setting of COVID‐19 (Table 4).^10^, ^157^, ^158^, ^159^, ^160^, ^161^, ^162^, ^163^, ^164^ These include patients with classic proximal myopathy,^10^, ^157^, ^158^, ^159^, ^164^ cases with marked bulbar involvement,^160^ presentations consistent with dermatomyositis,^161^ including cases with amyopathic dermatomyositis and interstitial lung disease.^165^, ^166^ In the majority of cases, the diagnosis of myositis was determined based on clinical presentation supported by laboratory findings (ie, CK elevation, and, when available, myositis‐specific autoantibodies) in the setting of a molecular diagnosis of viral (ie, SARS‐CoV‐2) infection. In few patients, the diagnosis was confirmed by muscle biopsy^10^, ^160^ and/or muscle MRI.^158^, ^160^, ^162^ In one case, electron microscopy ruled out direct viral invasions as pathophysiological mechanism of muscle damage.^160^ A dramatic case of limb ischemia complicated by severe muscle injury, inflammation and compartment syndrome was attributed to COVID‐19‐induced hypercoagulability.^163^

In a case series of COVID‐19‐associated paraspinal myositis,^162^ seven out of nine patients who underwent spine MRI for back pain, lower extremity weakness, or lower extremity paresthesia were found to have edema and enhancement of the paraspinal muscles (ie, erector spinae and multifidus paraspinal muscles) at the lumbar level. Although the clinical relevance of these finding was unclear, the authors hypothesized that myositis could be relatively common in COVID‐19 patients. It cannot be ruled out that the paraspinal involvement could have been secondary to a protracted immobilization among severe COVID‐19 patients.

The biopsy‐based series of 35 patients by Suh et al showed a high percentage of critically ill COVID‐19 patients with histological evidence of muscle involvement although, unfortunately, this was not supported by any information on clinical presentation and neurologic exam.^23^ Specifically necrotizing myopathy occurred in nine patients, as shown by myophagocytosis of necrotic fibers, whereas myositis occurred in seven cases and was characterized by perivascular and endomysial inflammatory cell infiltrates, which were mainly CD68‐positive, CD4‐positive, and/or CD8‐positive histiocytes and T‐cells. Diffuse or multifocal MHC‐1 immunostaining of non‐necrotic/non‐regenerating muscle fibers was evident in all 16 patients with myositis or necrotizing myopathy. In one case, MHC‐1 was positive in the perifascicular muscle fibers, a finding often seen in dermatomyositis; however, this was not supported by any clinical information. An autopsy series in Brazil found evidence of myositis in two out of 10 examined autopsies, although the study provided few clinical and histological data.^167^

The potential of COVID‐19 to cause new‐onset myasthenia gravis (MG) has been proposed based on few case reports of both ocular^168^ and generalized MG.^4^, ^169^, ^170^ One study described three patients without previous neurologic or autoimmune disorders who developed ptosis, diplopia, and/or dysphagia 5 to 7 days after COVID‐19.^169^ Patients were diagnosed based on decremental responses on repetitive nerve stimulation and positive acetylcholine receptor (AchR) antibody testing. Treatment with steroids, IVIG and PEX was effective. Few cases of anti‐muscle‐specific tyrosine kinase (MuSK) MG have been also described.^171^, ^172^ COVID‐19 could be a trigger of MG exacerbations, possibly with worse outcomes. We found literature reports on MG crisis requiring mechanical intubation, although the reported cases were responsive to IVIG.^173^, ^174^ One bias is that some of the medications that have been used for COVID‐19, such as hydroxychloroquine and azithromycin, may cause MG exacerbations. A further concern is that a superimposed infection with Sars‐CoV‐2 may worsen the respiratory status of patients admitted for MG exacerbation. A retrospective Brazilian study in 15 MG patients with COVID‐19 showed high invasive ventilation and mortality rates (73% and 30%, respectively).^175^ Smaller case series have confirmed the more frequent need for invasive ventilation, however with lower mortality rates.^174^, ^176^

MG patients may be more prone to be infected by SARS‐CoV‐2 given their autoimmune disorder or treatment‐related immunosuppression, and they may have worse outcomes due to the risk of neuromuscular respiratory failure. In the early stages of the pandemic, two major registry‐based studies focused on this topic.^177^, ^178^ Using the TriNetX COVID‐19 Research Network platform (www.trinetx.com), a global COVID‐19 dataset, Roy and colleagues found that MG patients with COVID‐19 had a significantly higher risk of hospitalization and death when compared to the entire COVID‐19 cohort.^177^ Data from the COVID‐19 Associated Risks and Effects in MG (CARE‐MG), a registry launched by a global MG working group demonstrated a mortality of 24% and MG relapse rate of 40% among 91 patients, which is higher than expected in non‐COVID‐19 MG.^178^ More recent retrospective studies, however, have shown that the risk of COVID‐19 in MG patients may not be higher than that of the general population,^179^ that COVID‐19 may affect only minimally the course of MG,^179^, ^180^, ^181^ and mainly in those with high Myasthenia Gravis Foundation of America (MGFA) class (ie, ≥IV).^180^

### 2.6 ICU‐related PNS complications

In one of the few available prospective COVID‐19 series, complications related to ICU stay represented the most common cause of PNS involvement, with critical illness myopathy (CIM) and/or critical illness polyneuropathy (CIP) being the most common diagnoses (eight out of nine patients with PNS involvement).^10^ The first published case series on CIM/CIP and COVID‐19‐reported 11 patients with clinical and neurophysiological diagnosis of CIM or CIP among 225 COVID‐19 patients admitted to ICU.^182^ This proportion was lower than pre‐COVID‐19 literature data, but likely affected by higher mortality rates related to COVID‐19 and/or inability to perform electrodiagnostic studies in all patients given the stress on the healthcare system. The available electrophysiological and biopsy data in this cohort did not show any distinctive feature. Of note, muscle biopsies did not show thrombi or inflammatory infiltrates in the vessels. A smaller (n = 6) but well‐characterized cohort of CIM patients was described by Madia et al, with patients initially suspected of having myopathy due to ventilator wean failure, 6 to 14 days from initial intubation.^183^ Patients presented clinically with flaccid quadriplegia and preserved cranial muscles, electrophysiological studies were consistent with irritable myopathy and preserved sensory responses, CK was normal or mildly elevated (highest level of 1274 UI/L), and correlated prospectively with the course of the disease, which was overall benign with complete or almost complete recovery. The majority of patients had received hydroxychloroquine, but no specific treatments were trialed for the concomitant myopathy.^183^ Based on prospective case series showing potential benefit of IVIG to prevent disease progression in COVID‐19^184^ and on similar experiences with influenza A and B infection, early administration of IVIG has been proposed as a potential preventative intervention for CIM/CI, although evidence is still limited to individual case reports.^185^

Prone positioning has been found beneficial in ARDS and successfully translated to the COVID‐19 care in the ICU, but it has also posed unexplored challenges.^186^ In the setting of PNS disease, entrapment neuropathies have been the most common complication.^187^, ^188^, ^189^, ^190^, ^191^ Among 83 patients admitted for COVID‐19‐related ARDS and requiring prone ventilation, 12 (14.5%) developed this complication.^187^ The most frequent sites of injury were ulnar nerve (28.6%), radial nerve (14.3%), sciatic nerve (14.3%), brachial plexus (9.5%), and median nerve (9.5%). A similar study in Italy involved 135 COVID‐19 patients requiring prone ventilation of whom 7 (5.2%) developed entrapment neuropathies, with again the ulnar nerve (five out of seven) and the brachial plexus (two out of seven) being the most frequently affected. In the majority of cases, axonotmesis was evident on neurophysiological exams.^188^ These percentages are higher than expected based on the clinical trials that have validated the use of prone ventilation in the pre‐COVID era.^186^ One hypothesis is that patients with COVID‐19 ARDS may be more vulnerable to peripheral nerve injury, but no control patients were included to address this question in both studies.^187^, ^188^ Long and repeated prone positioning, and the comorbidities associated with severe COVID‐19 (eg, diabetes, obesity, old age) rather than direct mechanisms could explain a predisposition to more frequent nerve injury among COVID‐19 patients.

Compression of the lateral femoral cutaneous nerve at the level of the anterior‐superior iliac spine or inguinal ligament may be a relatively uncommon but specific complication of prone positioning.^192^, ^193^ Additional complications have been linked to nerve injury during endotracheal tube insertion or as a result of its displacement during prone positioning. A case of Tapia syndrome (ie, concomitant paralysis of hypoglossal and vagus nerves) has been described in one COVID‐19 patient.^132^ At least one of the two cases of hypoglossal paralysis reported in Table 2 was likely due to orotracheal intubation and prone ventilation rather than to multineuritis as hypothesized.^133^

### 2.7 Long COVID and PNS involvement

Descriptions of COVID‐19 patients who develop neurological complaints for several months after the resolution of respiratory symptoms are increasingly reported. Interestingly, and probably a *unicum* in the history of neurology, the first reports of such nature have been spread by patients themselves, using social network platforms, such as Twitter and Facebook, and then amplified by the mainstream media.

The terms “long‐COVID” or post‐acute sequelae of SARS‐CoV‐2 (PASC)^194^ have been used to describe this picture, although there is no consensus on the potential timeline of disease progression, from acute (eg, less than 4 weeks), to subacute (eg, 4‐12 weeks) and chronic (eg, more than 12 weeks), since the natural history of the entity itself is unknown. This and other methodological flaws are major limitations to the interpretation of the literature on long‐COVID, including timing and type of assessment (self‐administered questionnaires, interviews, digital apps, physical assessment), poor definition of symptoms and inclusion criteria, heterogeneity of COVID‐19 severity, co‐morbid illnesses (either known or undiagnosed) with respective treatments and lack of control groups.^195^

Initial reports have focused on the involvement of the CNS, suggested by subtle symptoms and signs of cognitive and neuropsychological impairment, frequently described under the umbrella term of “brain fogginess,” such as mental slowness, memory difficulties, poor concentration, mental fatigue, and anxiety. Depending on the epidemiological scenarios, either hospitalized or self‐quarantined patients, the frequency of cognitive impairment 4 months after onset has been reported in up to 38% and 18% of patients, respectively.^196^, ^197^ These findings are supported by impaired performance on neuropsychological testing and by evidence of frontal and parietal hypometabolism on FDG‐PET.^198^, ^199^ Proposed mechanisms include long lasting neuronal damage caused by hypoxia, neuroinflammation, or virus permanence.^200^

More recently, an increasing body of literature has suggested an involvement of the PNS during the later stages of COVID‐19. As pointed above, anosmia and dysgeusia besides being common early COVID‐19 symptoms, seem to persist in up to 27% of patients after the acute phase, possibly suggesting irreversible damage to the nerve terminals or the sensory receptor cells.^197^

Pain is one of the most common long‐term PNS symptoms after COVID‐19, reported by up to 30% of patients depending on the cohorts.^200^ Localized pain, such as chest pain, joint pain, and headache is the most frequent complaint, but there is an increasing number of reports on a more diffuse and ill‐defined pain among long‐haulers, frequently associated with descriptors such as fatigue, myalgia, and paresthesia.^196^, ^197^, ^200^ One hypothesis is that the release of pro‐inflammatory cytokines during the acute infection may cause hypersensitization of peripheral nociceptors followed by plastic changes and central sensitization during the chronic stage.

Muscle atrophy seems to be an early feature of severe COVID‐19, in possible relation to the release of proinflammatory cytokines (TNF‐alpha, IL‐1 and IL‐6), a mechanism that has been well established in other diseases, such as AIDS and cancer, where muscle loss is a prominent symptom.^201^ Additional mechanisms, specific to COVID‐19, could be prolonged immobilization with type 2 muscle atrophy, use of high‐dose steroids and neuromuscular blockade, and nutritional deficiencies related to prolonged feeding assistance. Whether these manifestations are reversible and their long‐term impact on COVID‐19 patients remains to be determined, and prospective studies are still lacking.

Fatigue has been described in as high as 53% of patients at 2 months after resolution of other COVID‐19 symptoms.^202^ Its frequent association with symptoms, such as tachycardia, postural hypotension, dizziness, low‐grade fever, bowel, bladder, or sexual dysfunctions seems to support a role for autonomic dysfunction as a possible encompassing mechanism. A subset of these patients meets the criteria for postural orthostatic tachycardia syndrome (POTS). In two case series on patients presenting after COVID‐19 with fatigue and other lingering symptoms, such as palpitations, dizziness, or dyspnea, POTS was the final diagnosis in the majority of cases.^203^, ^204^, ^205^ A possible role of autonomic dysfunction was suggested by a case series of 50 outpatients presenting with chronic fatigue 3 months after COVID‐19, where 26% had sudomotor dysfunction as diagnosed by electrochemical skin conductance.^206^ In a comparable study on 27 patients referring to the Mayo Clinic for similar complaints, sudomotor function was abnormal in 36%, cardiovagal function in 27%, and cardiovascular adrenergic function in 7% of patients.^205^

Sensory symptoms referable to small fiber neuropathy (SFN) have been reported in a subset of long haulers. Abrams et al retrospectively studied the clinical features of 13 patients presenting with painful paresthesia and numbness that developed during or after SARS‐CoV‐2 infection and who had nerve conduction studies showing no evidence of a large fiber polyneuropathy.^207^ Six out of 13 patients had a final diagnosis of SFN on skin biopsy, including two cases with dysautonomia on autonomic testing.^207^ No correlation with COVID‐19 severity was found.^207^

The spectrum of symptoms associated with long COVID has prompted comparisons with myalgic encephalomyelitis or chronic fatigue syndrome (ME/CFS).^208^ This is not surprising as ME/CFS could be secondary to viral infections such as EBV, rotavirus, or HHV‐6, among others.^209^ One may hypothesize that at least some of the symptoms observed with long COVID could be a non‐specific response to an infectious (viral) illness in predisposed individuals, as it has been proposed for ME/CFS.^208^

Despite the mounting pressure from the public opinion, which parallels the increasing frequency of referrals to neurology for “long‐COVID” symptoms, the quality and quantity of literature on this topic is still limited. Many questions remain unanswered, including the temporal criteria for defining “long‐COVID” itself, whether this is a single entity or an umbrella category for multiple and unrelated presentations, and, more importantly, whether it is secondary to a non‐neurological pathological process, such as a psychiatric disorder (such as post‐traumatic stress disorder or depression), or expression of the pulmonary and/or cardiac involvement in the early stages of the disease.

### 2.8 COVID‐19 vaccines: An overview

To date, the Food and Drug Administration (FDA) has granted emergency use authorization for three COVID‐19 vaccines: two mRNA vaccines, that is, BNT‐162b2 SARS‐CoV‐2 vaccine (Pfizer/BioNTech)^210^ and mRNA‐1273 SARS‐CoV‐2 vaccine (Moderna),^211^ and one replication‐deficient adenovirus‐based vaccine, that is, Ad26.COV2.S (Johnson & Johnson).^212^ Booster doses have been recently approved for these vaccines as well. Two other adenovirus‐based vaccines, ChAdOx1 nCoV‐19 vaccine (AstraZeneca/Oxford or Vaxzevria)^213^ and COVID‐Vac/Sputnik V (Gamaleya Institute)^214^ have been granted conditional marketing authorization in Europe and Russia. An additional vaccine based on a radically innovative approach, that is, recombinant protein nanoparticles, NVX‐CoV2373 (Novavax), is at and advanced stage of development, with a phase III clinical trial showing efficacy and safety rates similar to the mRNA vaccines.^215^ For multiple other vaccine candidates (https://covid19.trackvaccines.org/vaccines/), some of which already available for use in different countries, to date no large phase three clinical trials have been published. Among these, BBIBP‐CorV vaccine (Sinopharm, Beijing Bio‐Institute of Biological Products Co. Ltd.),^216^ an inactivated SARS‐CoV‐2 isolate, has been listed for emergency use by the World Health Organization (WHO) (https://www.who.int/news/item/07-05-2021-who-lists-additional-covid-19-vaccine-for-emergency-use-and-issues-interim-policy-recommendations), potentially expediting its global roll out.

An overwhelming amount of both retrospective and prospective data have suggested that COVID‐19 vaccines are safe, and beside decreasing COVID‐19‐associated morbidity and mortality, vaccination has been associated with lower mortality rates from all other causes, supporting the notion that COVID‐19 vaccination does not increase the risk of death.^217^

### 2.9 Guillain‐Barré syndrome following COVID‐19 vaccines

Concern for autoimmunity secondary to COVID‐19 vaccines has been raised by the adenovirus vaccine trials, with cases of transverse myelitis reported after the Johnson & Johnson and AstraZeneca vaccines, although later determined to be unlikely related to the vaccine.^212^, ^213^ Similar concerns of have been raised by the mRNA vaccines as well, Pfizer and Moderna, which have been associated with higher than expected rates of myocarditis and pericarditis in post‐marketing surveillance studies and real‐world cohorts.^218^, ^219^

The potential of COVID‐19 vaccines to cause GBS, particularly the adenovirus vector‐based, vaccines has been initially suggested by cases that occurred during the phase III clinical trials. The association between GBS and vaccination has been long debated since initial reports of increased incidence of GBS after the swine influenza vaccine during the USA/New Jersey 1976 vaccination campaign.^104^ Thereafter, similar concerns have been raised for multiple vaccines, including oral polio, DPT, rabies, hepatitis B, and quadrivalent conjugated meningococcal vaccines.^220^ However, large case‐control studies have failed to show causal association.^220^ Two patients in the Johnson & Johnson trial developed GBS after the single‐dose vaccine injection, specifically one in the placebo group and one in the vaccine group, thus, with identical incidence in both the trial arms.^221^ The case in the vaccine arm was an otherwise healthy 60‐year‐old female who developed a GBS/MFS overlap syndrome 10 days after the vaccine administration. No distinctive features were noted, and she was negative for anti‐GQ1b antibody.^221^ No GBS case occurred during the AstraZeneca phase III clinical trial. In addition, there have been no reports of GBS after the administration of mRNA vaccine during the clinical trials.

Following the start of the worldwide mass vaccination campaign, real‐world case reports of GBS after COVID‐19 vaccine have emerged. Until 1 December 2021, we identified a total of 24 case reports and 16 publications,^221^, ^222^, ^223^, ^224^, ^225^, ^226^, ^227^, ^228^, ^229^, ^230^, ^231^, ^232^, ^233^, ^234^, ^235^, ^236^ with the majority of cases occurring after the AstraZeneca vaccine (17 out of 24).^222^, ^224^, ^225^, ^229^, ^230^, ^231^, ^232^, ^235^, ^236^ Initial reports included two patients with classic AIDP occurring 11 and 14 days after the first dose of the ChAdOx1c nCoV‐19 vaccine (AstraZeneca).^224^, ^225^ GBS variants, including bifacial weakness with or without paresthesia, and pure sensory GBS, could be more common after the AstraZeneca vaccine, occurring in 13 out of 17 cases. All cases occurred after the first dose, with a median time from vaccination to symptom onset of 11 days (range 7‐21 days). The neurophysiological diagnosis was AIDP in the majority of cases (13 out of 14 cases where it was available), whereas in a single case it was AMSAN. Patients were treated with conventional therapies (ie, IVIG) and the outcome was positive, with GBS disability score ≤ 2 in 10 out of 14 cases where this information was available. The occurrence of GBS after mRNA‐based vaccines has been also reported, although less commonly (n = 5 cases).^223^, ^227^, ^228^, ^233^, ^234^ Interestingly, GBS occurred after the second dose as well (three out of five cases), with a range of 5 to 16 days, as well as after the first with a range of 1 to 14 days. The clinical presentation was paraparetic GBS in four out of five cases, with the remaining case being a classic form. Neurophysiological studies were consistent with AIDP in two cases and AMSAN in one case. Beside the GBS case during the Johnson & Johnson trial,^221^ we did not find other reports associated with this vaccine. The literature on GBS after the other COVID‐19 vaccines is quite scarce, with to date a single report of classic GBS (AMSAN) 5 days after the second dose of the CoronaVac vaccine.^226^

A cohort study conducted from 1 January 2021 to 30 June 2021 at the Birmingham University Hospital, United Kingdom, compared 16 cases of GBS presenting within 4 weeks after the first COVID‐19 vaccine (14 had received the AstraZeneca vaccine and the remaining two the Pfizer and Moderna vaccines) to a historical cohort of 114 consecutive GBS patients diagnosed between 2005 and 2019.^237^ The authors found a 2.6‐fold increase in number of admissions for GBS during the study period, compared to the same period in the previous 3 years.^237^ Patients presenting with GBS after AstraZeneca vaccine had more frequent facial and bulbar involvement than the historical cases, and more commonly they had the bifacial weakness and distal paresthesia GBS variant, similar to the above‐mentioned reports.^237^, ^238^, ^239^

Large surveillance programs to identify any excess of GBS cases after any of the vaccines are already underway, including initiatives from national and international public health agencies and institutions (CDC, FDA, EMA, WHO), and neurological societies such as the Peripheral Nerve Society (www.pnsociety.com) and the International GBS Outcome Study group (https://gbsstudies.erasmusmc.nl/). Based on preliminary reports, in the United States, there have been 132 cases of GBS after 13.2 million doses of Ad26.COV2.S vaccine (Johnson & Johnson).^240^ The estimated rate is 9.8 cases per million doses, that is approximately four times the expected rate. The median age is 56 years (range 45‐62 years), the median time to onset from vaccination is 13 days, 35% had a life‐threatening presentation, and one patient died.^240^ In a preliminary report on 100 cases from the same series, and unexpectedly high frequency of bifacial weakness, in up to 25% of patients, was reported. In Europe, a total of 227 cases of GBS occurred after 51 million doses of ChAdOx1 nCoV‐19 (AstraZeneca) (https://www.ema.europa.eu/en/documents/covid-19-vaccine-safety-update/covid-19-vaccine-safety-update-vaxzevria-previously-covid-19-vaccine-astrazeneca-9-december-2021_en.pdf). Based on these data, the European Medicines' Agency (EMA) safety committee has recommended a change in product information for the AstraZeneca vaccine to include a warning about cases of GBS reported following vaccination (https://www.ema.europa.eu/en/news/meeting-highlights-pharmacovigilance-risk-assessment-committee-prac-5-8-july-2021). Data derived from the English National Immunization Database of SARS‐CoV2 vaccinations linked to hospital admission data, found a 2.04‐fold increased risk for GBS (95% confidence interval [CI]: 1.60‐2.60) within 28 days after the AstraZeneca vaccine administration, but not after the Pfizer vaccine.^241^ In three districts of India, over the 1.5 million individuals who were vaccinated with COVID‐19 vaccines between mid‐March to mid‐April 2021, with 80% being ChAdOx1‐S nCoV‐19 (AstraZeneca), there were seven cases of GBS that occurred within 2 weeks of the first dose of vaccination.^239^ All seven patients developed severe GBS, and a higher than expected rate of bilateral facial weakness was observed. The frequency of GBS was calculated to be 1.4‐ to 10‐fold higher than expected in that population and in the same period of time. In a large multi‐institutional study in Taiwan involving 18 269 healthcare workers who received AstraZeneca vaccine between 22 March and 31 May 2021, one single case of GBS variant (bilateral facial palsy with paresthesia) after the first vaccine dose was identified.^242^ A similar large cohort study in Mexico among 3 890 250 recipients of the Pfizer vaccine within 30 days from the first vaccine administration, identified seven incident cases, with observed incidence of 0.18/100 000 which was similar to the expected community‐based rate, indicating no increased risk.^243^ Among the 613 780 patients who had received both doses, no GBS cases were reported. Of note, the seven GBS cases occurred after a median of 6 days (range 3‐28), were classic GBS in the majority of cases (five out seven), with a relatively higher proportion of AMAN (four out of seven) as would be expected in this geographical scenario.^243^

In contrast, during surveillance studies for the mRNA‐based vaccines, no vaccine‐outcome association, including the occurrence of GBS, met the pre‐specified requirement for a signal.^244^ This may suggest that, similar to thrombotic complications (see below), antigens mimicking neural components may be related to the structure of the adenovirus vectors, and this would explain the relative safety of mRNA vaccines.^245^

Overall, these data are preliminary and should be taken cautiously, without leaping to costly conclusions.^246^ The only cohort study so far on GBS and COVID‐19 vaccines is retrospective, included four cases out of 16 that later were diagnosed has having acute onset CIDP, was small and could have been affected by random clustering bias.^237^ Surveillance programs have so far been limited to relatively short periods of time (weeks or months). The surveillance system itself is based on passive reporting, which is subject to under‐reporting and lack of direct and unbiased comparison of groups. Spontaneous reporting frequently contains incomplete medical record information (ie, clinical findings, electrodiagnostic studies, CSF data, diagnostic certainty, response to treatment), and therefore GBS cases must be considered presumptive pending analysis of medical records and definitive diagnoses. Finally, these preliminary analyses compared the observed GBS incidence with expected rates reported in the pre‐COVID literature, but this assumes that the vaccinated population is subject to the same background rate as the population that was assessed in the literature.

Assuming an incidence of 8 to 19 GBS cases/million adults/year,^247^ for every billion people vaccinated against COVID‐19, by chance alone we would expect to see 900 to 2200 GBS cases within 6 weeks after a one dose vaccine, and 1500 to 3700 within a 10‐week period after a two‐dose vaccine.

### 2.10 COVID‐19 vaccines and cranial neuropathies

The occurrence of cranial neuropathies after COVID‐19 vaccines is increasingly being reported following initial reports of Bell's palsy during the phase III clinical trials of both mRNA vaccines. Specifically, during the Pfizer‐BioNTech clinical trial, which included 43 448 participants (21 720 BNT162b2 vs 21 728 placebo), four patients diagnosed with Bell's palsy were reported (0.018%) in the vaccine group and none in the placebo group (day 37 after dose 1 and days 3, 9, and 48 after dose 2).^210^ The Moderna trial, which enrolled 30 420 volunteers (15 210 participants in each group), reported three cases in the vaccine (0.02%) and one in the placebo group (0.007) (22, 28, and 32 days after dose 2).^211^ All except one case occurred after the second dose, with a median delay of 25 days (range 3‐48). These data are relative to a median follow‐up of two months. As pointed out by Oznoff et al,^248^ if expressed as number of cases per 100 000 person‐years, the observed incidence of Bell's palsy in the 40 000 combined vaccine arm participants was 3.5‐7‐times higher than it would be expected in the general population. Associations between vaccines and Bell's palsy have been reported in the past, particularly with the H1N1 influenza vaccine,^248^ but of note they were all protein‐based vaccines and/or contained an exogenous protein adjuvant. New and yet unknown mechanisms may be responsible for this complication with mRNA vaccines. Based on such evidence, the FDA has recommended post‐marketing surveillance for cases of Bell's palsy in the general population. The first real‐world report^249^ described a 37‐year‐old otherwise healthy male developing Bell's palsy 15 days after the first dose of the Pfizer vaccine. In an additional report, a patient with a history of recurrent idiopathic facial palsy (three prior episodes over 8 years), developed a new episode of Bell's palsy 36 hours after the administration of the second dose of the Pfizer vaccine.^250^ A report from the United Kingdom described recurrent and side‐changing facial nerve palsy occurring shortly after each dose of the Pfizer‐BioNTech vaccine: the first episode involved the left facial nerve and occurred 5 hours after administration of the first vaccine dose.^251^ Six weeks later, after a complete recovery with prednisolone treatment, the patient received the second dose, and 2 days later he developed a more severe (House‐Brackmann grade 4) right‐side Bell's palsy, with incomplete response to high dose steroids. Of note, this patient had type 2 diabetes and multiple vascular risk factors.^251^ In Israel, that has one of highest world pro‐capita vaccination rates, a case series of nine patients experiencing Bell's palsy after the Pfizer vaccine was published.^252^ Interestingly, in six out of nine cases, it occurred after the first dose (median 6 days, range 3‐11) and not after the second one as reported during the clinical trials.^252^ Post‐vaccine monitoring so far has not identified an association between COVID‐19 vaccination and Bell's palsy (https://www.cdc.gov/vaccines/acip/meetings/downloads/slides‐2021‐01/06‐COVID‐Shimabukuro.pdf).

Isolated cranial neuropathies, including optic nerve,^253^ oculomotor nerve,^253^ and abducens nerve^253^, ^254^, ^255^ have been reported after adenovirus‐based vaccines (6‐30 days after the first dose)^253^ and the Pfizer vaccine (2 days after the first dose).^254^ Brain and orbit MRI with contrast did not show gadolinium enhancement when such study was available.^253^, ^254^

Multiple cranial neuropathies, specifically ipsilateral oculomotor, abducens, trigeminal, and facial palsy, were reported 6 days after the first dose of the Pfizer‐BioNTech COVID‐19 vaccine.^256^ Brain MRI with contrast revealed enhancement in the clinically affected cranial nerves, whereas CSF studies and additional differential diagnostic laboratory tests were negative. The patient responded to high doses of i.v. corticosteroids.^256^ An additional report of acute bilateral oculomotor nerve palsy, likely overlapping with an incomplete variant of MFS (ie, acute ophtalmoparesis), has been reported 18 days after the first dose of Pfizer vaccine.^257^ Given the overlapping presentation with MFS, the patient was treated with IVIG with complete response. CSF and neurophysiological studies, and anti Gq1b testing were all negative.^257^

As of 2014, only four cases of cranial palsies excluding the facial nerve have been reported by the US Vaccine Adverse Event Reporting System (VAERS) after inoculation of a large number of non‐COVID‐19 vaccines, both inactivated and live attenuated.^258^ A similar effort is undergoing for COVID‐19 vaccines as well.

### 2.11 COVID‐19 vaccines, CIDP, and other neuropathies

In our review, we found isolated reports of “acute onset” CIDP following Moderna^259^ and AstraZeneca vaccines,^235^, ^260^ in all cases 3 weeks after the first dose. Of note, one of the cases had a similar presentation years prior after the influenza vaccine, but he was asymptomatic since then.^259^ All cases had a good recovery after standard treatment. In the UK cohort on GBS within 4 weeks after COVID‐19 vaccines, four out of 16 patients were diagnosed with acute‐onset CIDP.^237^ No clinical or neurophysiological exams are available for these patients, besides the fact that two were subsequently re‐treated with IVIG, one patient with corticosteroids, and one with plasma exchanges with good outcome.^237^

In the pre‐COVID era, the Italian CIDP database identified vaccination as the anteceding event in 1.5% of 411 patients 1 to 42 days before the diagnosis of CIDP.^261^ Given the chronic nature of CIDP and the fact that its diagnosis requires a progression over 8 weeks, attributing its onset to a single event (ie, vaccination) is challenging. As an example, one of the post‐COVID‐19 vaccine CIDP patients had also received the influenza vaccine 6 weeks prior to the onset of symptoms, while the COVID‐19 vaccine had been administered 3 weeks prior.^235^ When reviewing the potential of COVID‐19 vaccines to exacerbate or worsen CIDP in patients with an established diagnosis, we did not find any report so far. Our current knowledge on the use of vaccines of any kind in patients with a prior CIDP diagnosis is quite limited. Three cohort studies have tried to address this question,^262^, ^263^, ^264^ reaching contrasting conclusions due to significant methodological differences and limitations.^265^

ChadOx1 nCoV‐19/AZD1222 (AstraZeneca) and Ad26.COV2.S (Johnson & Johnson) vaccines, but not the mRNA‐based vaccines, have each been associated in real‐world reports with a small risk of thrombotic events, pathophysiologically similar to heparin‐induced thrombocytopenia (HIT). Reported cases included cerebral venous sinus thrombosis.^266^ To date no PNS complication ascribable to a hypercoagulable state secondary to the adenovirus‐based vaccines has been reported.

Several reports of neuralgic amyotrophy or Parsonage‐Turner Syndrome after COVID‐19 vaccines are emerging, interestingly after both mRNA^267^, ^268^, ^269^, ^270^, ^271^ and adenovirus‐based vaccines.^270^, ^272^, ^273^, ^274^ As expected, most patients presented with paralysis preceded by pain, although with some exceptions^271^ (well known to the pre‐COVID literature). Most cases involved the brachial plexus and, less frequently, the lumbosacral plexus.^272^ Interestingly, one patient complained of onset of pain around the injection site that spread to the shoulder and the arm.^270^ Overall, the incidence of post‐vaccination Parsonage‐Turner syndrome seems very low. The most reliable reference is the influenza vaccination campaign, with only 18 cases reported in the Vaccine Adverse Effect Reporting System from 2018 to 2020 (http://wonder.cdc.gov/vaers.html).

### 2.12 COVID‐19 vaccines and muscle involvement

Myalgia is a common adverse effect of COVID‐19 vaccines, occurring in up to 50% of recipients in the Moderna trial after the second dose^211^ and up to 60% in the single‐dose Johnson & Johnson trial.^212^ It is reported as transient and occurring along with systemic symptoms like fever, headache, arthralgia, and fatigue. Similar flu‐like symptoms were reported in the Vac/Sputnik V vaccine trial, although in only 5% of cases.^214^ Among 1.6 million Pfizer vaccine recipients in the United States who responded to a post‐vaccination survey, 17% and 37% of patients reported myalgias after the first and second dose, respectively, whereas fevers, chills, and joint pain each occurred in approximately 20% of cases.^275^ Similarly, for the Moderna vaccine recipients during post‐marketing surveys, myalgias were reported in 21% and 51% after the first and second dose, respectively, while fever/chills and joint pain occurred in approximately 40% and 32% of nearly 2 million responders.^275^

Clinical and laboratory diagnoses of rhabdomyolysis have been reported with both mRNA vaccines, after the first and second dose.^276^, ^277^ Reports of localized muscle inflammation at the injection site of COVID‐19 vaccine, as demonstrated by MRI or biopsy, have been reported as well,^278^, ^279^ and represent site reaction rather than systemic disease. So far, we found a single report of myositis after the Moderna vaccine, with involvement of the proximal lower extremities and extensions to the fascia.^280^ This case had a complete response to i.v. steroids.^280^

## 3 CONCLUSION

As we write, the body of literature linking COVID‐19 and vaccines to PNS complications increases daily. Moreover, several other cases might have been observed worldwide and never been published for different reasons. As we move from the pandemic to the vaccine era, a number of questions remain unanswered, including: (a) whether the risk of PNS complications among breakthrough infections after vaccination could be higher, potentially driven by higher degrees of immune‐mediated responses (and, potentially, of molecular mimicry), although available literature suggests that COVID‐19 seems to be milder among vaccinated patients;^281^ (b) whether the emergence of SARS‐CoV‐2 variants, such as the Beta and the Omicron variants, could select strands with more marked neurotropism and direct PNS invasion capabilities, similar to SARS‐CoV and MERS‐CoV; (c) whether booster vaccinations and the combination of different types of vaccines in this setting could cause more frequent PNS complications, although preliminary reports indicate this may not be the case (https://www.cdc.gov/vaccines/acip/meetings/downloads/slides-2021-11-19/04-COVID-Shimabukuro-508.pdf).

Criteria for assessing causality between a proposed clinical outcome and a possible pathological insult were originally proposed by Austin Bradford Hill in 1965 and consist of nine characteristics: strength, consistency, specificity, temporality, biologic gradient, plausibility, coherence, experiment, and analogy.^282^ So far temporality and, possibly, plausibility seem to be the only criteria met by the conditions reviewed in this paper. Therefore, based on available data, any conclusion about a pathophysiological correlation between COVID‐19, vaccines and PNS disorders remains premature, while epidemiological, clinical and pathological data are insufficient.^283^

The occurrence of PNS complication after COVID‐19 vaccines seems very rare and limited to a possible higher risk of facial nerve palsy and possibly GBS, however, in a range that should not raise any concern on the need to pursue the vaccination campaign. Based on experiences with other vaccination campaigns and data coming from adverse monitoring systems, there is widespread consensus that the benefits of vaccination outweigh the risks related to adverse events. Although large cohort studies are still lacking, there is early evidence that the administration of COVID‐19 vaccines, specifically the Pfizer vaccine, among patients with known history of GBS is not associated with a significant risk of relapse.^284^ As such, multiple institutions including the CDC (https://www.cdc.gov/vaccines/hcp/acip-recs/general-recs/index.html), the American Association of Neuromuscular and Electrodiagnostic Medicine (AANEM),^285^ and an ad hoc group from the Peripheral Nerve Society^286^ encourage all patients with PNS disorders to adhere to the vaccination campaign, including those with history of CIDP, GBS, or MG.

## CONFLICT OF INTEREST

The authors declare no potential conflict of interest.
